# Dynamic monitoring revealed a slightly prolonged waiting time for total gastrectomy during the COVID-19 pandemic without increasing the short-term complications

**DOI:** 10.3389/fonc.2022.944602

**Published:** 2022-08-31

**Authors:** Xiaohao Zheng, Shikang Ding, Ming Wu, Chunyang Sun, Yunzi Wu, Shenghui Wang, Yongxing Du, Lin Yang, Liyan Xue, Bingzhi Wang, Chengfeng Wang, Wei Cui, Yibin Xie

**Affiliations:** ^1^ Department of Pancreatic and Gastric Surgery, National Cancer Center/National Clinical Research Center for Cancer/Cancer Hospital, Chinese Academy of Medical Sciences and Peking Union Medical College, Beijing, China; ^2^ Department of Gastrointestinal Surgery, Yun Cheng Center Hospital, Yuncheng, China; ^3^ Department of General Surgery, The Central Hospital of Jia Mu Si City, Jiamusi, China; ^4^ Department of General Surgery, Civil Aviation General Hospital, Beijing, China; ^5^ Department of Medical Oncology, National Cancer Center/National Clinical Research Center for Cancer/Cancer Hospital, Chinese Academy of Medical Sciences and Peking Union Medical College, Beijing, China; ^6^ Department of Pathology, National Cancer Center/National Clinical Research Center for Cancer/Cancer Hospital, Chinese Academy of Medical Sciences and Peking Union Medical College, Beijing, China; ^7^ State Key Lab of Molecular Oncology, National Cancer Center/National Clinical Research Center for Cancer/Cancer Hospital, Chinese Academy of Medical Sciences and Peking Union Medical College, Beijing, China; ^8^ Department of Clinical Laboratory, National Cancer Center/National Clinical Research Center for Cancer/Cancer Hospital, Chinese Academy of Medical Sciences and Peking Union Medical College, Beijing, China; ^9^ Department of Pancreatic and Gastric Surgery, National Cancer Center/National Clinical Research Center for Cancer/Hebei Cancer Hospital, Chinese Academy of Medical Sciences, Langfang, China

**Keywords:** gastric cancer, total gastrectomy, waiting time, COVID-19, SARS-CoV-2, Clavien-Dindo, complications, laboratory results

## Abstract

We aimed to determine the pattern of delay and its effect on the short-term outcomes of total gastrectomy before and during the coronavirus disease 2019 (COVID-19) pandemic. Overlaid line graphs were used to visualize the dynamic changes in the severity of the pandemic, number of gastric cancer patients, and waiting time for a total gastrectomy. We observed a slightly longer waiting time during the pandemic (median: 28.00 days, interquartile range: 22.00–34.75) than before the pandemic (median: 25.00 days, interquartile range: 18.00–34.00; *p* = 0.0071). Moreover, we study the effect of delayed surgery (waiting time > 30 days) on short-term outcomes using postoperative complications, extreme value of laboratory results, and postoperative stay. In patients who had longer waiting times, we did not observe worse short-term complication rates (grade II–IV: 15% vs. 19%, *p* = 0.27; grade III–IV: 7.3% vs. 9.2%, *p* = 0.51, the short waiting group vs. the prolonged waiting group) or a higher risk of a longer POD (univariable: OR 1.09, 95% CI 0.80–1.49, p = 0.59; multivariable: OR 1.10, 95% CI 0.78–1.55, p = 0.59). Patients in the short waiting group, rather than in the delayed surgery group, had an increased risk of bleeding in analyses of laboratory results (plasma prothrombin activity, hemoglobin, and hematocrit). A slightly prolonged preoperative waiting time during COVID-19 pandemic might not influence the short-term outcomes of patients who underwent total gastrectomy.

## 1 Introduction

A prolonged waiting time for surgery is a major concern for cancer patients and their relatives, as it may result in a deterioration of the patient’s condition, increase the risk of metastasis, and worsen anxiety (1, 2). Previous studies have reported that surgical delay in colorectal and breast cancer is associated with shorter overall survival (3). However, in patients with esophageal, pancreatic, or lung cancer, a delay in surgery seemed not have a worse clinical outcome (4, 5). Previously, the admission patterns of cancer patients were roughly associated with accompanied diseases needing intervention in advance, holiday and weekend admission, and bed turnover issues. As all known, the pandemic has dramatically changed cancer therapy admission patterns since the beginning of 2020 (6, 7). Disruption of typical preoperative assessments and referrals from local hospitals during the pandemic significantly increased preoperative waiting times for cancer patients (8). Moreover, the impact of the above-mentioned factors varied according to the local pandemic severity and its prevention policy, which may have added to the complexities of increased waiting time.

According to the guidelines, urgent surgery should be performed for aggressive tumors within 30 days of diagnosis. However, the effects of a slightly prolonged waiting time remain unknown. Unfortunately, during the coronavirus disease 2019 (COVID-19) pandemic, this has become an area of concern. The risk surplus of 30 days varied across different types of cancers during the COVID-19 period. The hazard ratio for each week of delay ranged from 1.06 to 1.08 in patients with prostate and breast cancer (9). Gastric cancer is the third most common cause of cancer-related death, with a high incidence and resultant mortality rate in China and worldwide (10, 11). Compared to partial gastrectomy, total gastrectomy (TG) is associated with a higher risk of metastasis as patients who require a TG have a higher pathological stage, larger tumor, or a worse gross type with a poor prognosis, such as linitis plastica. Furthermore, extensive invasion of the gastroesophageal junction results in extended resection of abdominal segment of esophagus, which is used to achieve a negative margin and can require conversion to a thoracotomy. However, it is unclear whether a slight delay leads to a significantly higher complication rate and longer hospital stay. In the coming years, a prolonged waiting time for TG is expected (8). However, the effect of a slightly protracted preoperative waiting time on the short-term prognosis of patients with gastric cancer remains undetermined. To investigate and compare the impact of the increase in waiting times for TG between the pre-COVID-19 and COVID-19 periods, we studied the dynamic changes in the admission patterns of patients who underwent TG. The complications, postoperative stay, and extreme postoperative laboratory test results of gastric cancer patients who underwent TG were used to compare the short-term outcomes between the short and delayed surgery groups in this study.

## 2 Methods and materials

### 2.1 Patient cohort

**Figure 1:**
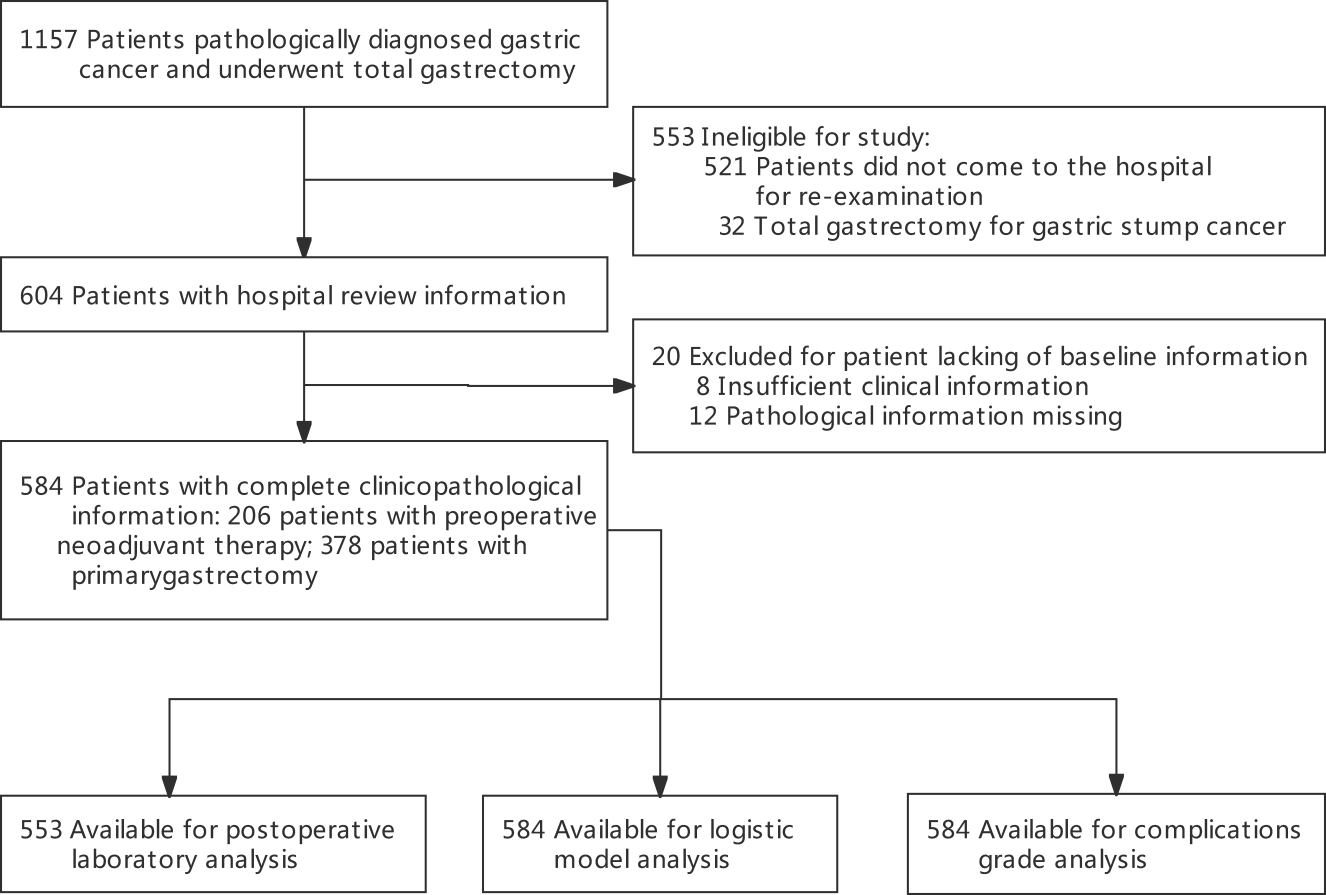
Flow chart.

In total, 1,157 patients with gastric cancer were treated with TG at the Cancer Hospital, Chinese Academy of Medical Sciences, China, between June 2014 and June 2021 (**Figure 1**). We included 584 patients who returned for re-examination at least once after undergoing TG. Data were collected from the prospectively documented electronic medical records at the National Cancer Center, China. The inclusion criteria for this study were: radical TG with D2 lymph node dissection, pathologically diagnosed primary gastric adenocarcinoma, and no other malignant tumor history. Patients were excluded from the study if they were lost to follow-up or the interval between the latest re-examination and TG was less than three months. Thus, our cohort did not include patients with 90-day postoperative mortality. This study was approved by the Research Ethics Committee of the Peking Union Medical College, and the approving body waived the need for informed consent due to the study’s retrospective nature.

### 2.2 Treatment procedure

The treatment methods for all patients were determined by a multidisciplinary treatment (MDT) group according to the results of the preoperative assessment. Surgery-alone (SA) patients received TG alone without neoadjuvant therapy, while neoadjuvant patients received neoadjuvant therapy until the tumor was clinically resectable, followed by TG, according to the guidance of the MDT team. The margins of resection and the extent of the lymph node dissection were based on the treatment guidelines issued by the Japanese Gastric Cancer Association (JCGA). Pathological examinations, which included the determination of the pathological type, stage, and immunohistochemical characteristics, were independently reported by two professional pathologists according to the definition of the eighth edition of the American Joint Committee on Cancer (AJCC) staging systems. Postoperative adjuvant therapy was recommended for all patients with advanced pathological stages, and the attending physician determined the specific chemotherapy.

### 2.3 Definition of preoperative waiting days and primary endpoint

To study the pattern of preoperative waiting time before and after the pandemic, patients were categorized into two periods depending on their admission date: before or after January 1^st^, 2020. To study the impact of delay, patients were categorized into two groups depending on their waiting time: delayed surgery (> 30 days) and short waiting time (≤ 30 days) groups. Waiting time was defined as the interval between the pathological diagnosis and TG for the SA patients and between the cessation of neoadjuvant therapy and TG for the neoadjuvant patients. The main research objective was the short-term postoperative prognosis, including the incidence of short-term postoperative complications defined by the Clavien-Dindo (CD) classification from the medical records, extreme laboratory results, and postoperative days (POD). POD > 14 days was defined as a “delay in discharge.”

### 2.4 Statistical analyses

Where appropriate, comparisons between the two groups concerning binomial outcomes were performed using the chi-square test and Fisher’s exact test. Nonparametric data with multiple comparisons were analyzed using the Kruskal–Wallis analysis of variance. The Mann–Whitney U test was used to compare the two groups. Data with a normal distribution were analyzed *via* analysis of variance (ANOVA) for multiple comparisons and Student’s t-test for comparisons between the two groups. Univariate and multivariate logistic regression were used to analyze the association among the tumor, patient characteristics, and overall and severe postoperative complications of CD grades II–IV and III–IV, respectively. The multivariable-adjusted model included pre-specified potential confounders such as sex, age, tumor histology, clinical T-stage, and clinical N-stage. R version 4.1 (www.r-project.org) was used for all the statistical analyses. All tests were two-sided, and statistical significance was set at *p* < 0.05. The minimum and maximum postoperative laboratory test results were recorded. Laboratory test results received log10 (original values +1) transformation regardless of units, whereas the sva package function Combat was used to remove the batch effect of laboratory items if the detection method changed over the years (12, 13).

## 3 Results

### 3.1 The COVID-19 pandemic has resulted in prolonged preoperative waiting times for TG patients with gastric cancer

Data of 1,157 patients in the National Cancer Center who were diagnosed with gastric cancer and underwent TG between June 2014 and May 2021 were extracted. Of these, 553 were ineligible based on the exclusion criteria, and 20 patients lacked baseline information (n = 20) were also excluded. Hence, the final study cohort consisted of 584 eligible patients (**Figure 1**) who were eligible for the study. The demographic and pathological characteristics of the patients are shown in **Table 1**. The mean (SD) age was 56.5 (10.9) years, and 20.5% were aged ≥ 65 years. A total of 31.7% had a history of accompanying diseases, including cardiopulmonary disease or diabetes. Approximately 35.3% of the patients received neoadjuvant therapy, while 64.7% received TG alone. Patients were classified into 2-year intervals (2014-2015, 2016-2017, 2018-2019, 2020-2021). We found no significant differences among the four time-period groups with respect to baseline clinical and pathological characteristics.

**Table 1:** Clinicopathological baseline characteristics of the 583 eligible patients who underwent total gastrectomy.

	2014-2015	2016-2017	2018-2019	2020-2021	P-values
**Total**	147	163	192	82	
**COVID-19 cases**	0(0.0)	0(0.0)	0(0.0)	0(0.0)	
**Age, mean (SD)**	55.53 (11.89)	56.13 (8.96)	57.02 (11.38)	57.55 (11.49)	0.469
**Age**					0.039
<65	117 (79.6)	140 (85.9)	149 (77.6)	58 (70.7)	
≥65	30 (20.4)	23 (14.1)	43 (22.4)	24 (29.3)	
**Sex**					0.449
Male	101 (68.7)	114 (69.9)	120 (62.5)	56 (68.3)	
Female	46 (31.3)	49 (30.1)	72 (37.5)	26 (31.7)	
**Smoking**					0.233
No	80 (54.4)	107 (65.6)	118 (61.5)	48 (58.5)	
Yes	67 (45.6)	56 (34.4)	74 (38.5)	34 (41.5)	
**Drinking**					0.624
No	81 (55.1)	101 (62.0)	117 (60.9)	49 (59.8)	
Yes	66 (44.9)	62 (38.0)	75 (39.1)	33 (40.2)	
**Diseases**					0.504
No	103 (70.1)	109 (66.9)	136 (70.8)	51 (62.2)	
Yes	44 (29.9)	54 (33.1)	56 (29.2)	31 (37.8)	
**Neoadjuvant therapy**					0.134
Yes	46 (31.3)	61 (37.4)	62 (32.3)	37 (45.1)	
No	101 (68.7)	102 (62.6)	130 (67.7)	45 (54.9)	
**Tumor Location**					0.005
Middle/Lower	98 (66.7)	100 (61.3)	113 (58.9)	35 (42.7)	
Upper	49 (33.3)	63 (38.7)	79 (41.1)	47 (57.3)	
**Size, mean (SD)**	6.11 (3.65)	5.81 (3.57)	6.19 (3.26)	5.71 (2.93)	0.609
**Lauren type**					0.136
Intestinal	22 (16.1)	37 (25.7)	50 (28.1)	17 (21.5)	
Mixed	39 (28.5)	39 (27.1)	41 (23.0)	15 (19.0)	
Diffuse	76 (55.5)	68 (47.2)	87 (48.9)	47 (59.5)	
**Bormann type**					0.748
0-1	27 (21.4)	23 (18.1)	27 (16.8)	13 (16.7)	
2-4	99 (78.6)	104 (81.9)	134 (83.2)	65 (83.3)	
**Differentiation**					0.208
Poorly differentiated	131 (89.1)	135 (82.8)	172 (89.6)	73 (89.0)	
Well differentiated	16 (10.9)	28 (17.2)	20 (10.4)	9 (11.0)	
**Vessel invasion**					0.087
Negative	61 (45.5)	76 (46.6)	85 (44.3)	25 (30.5)	
Positive	73 (54.5)	87 (53.4)	107 (55.7)	57 (69.5)	
**Nerve invasion**					0.051
Negative	45 (32.6)	50 (30.7)	53 (27.6)	13 (16.0)	
Positive	93 (67.4)	113 (69.3)	139 (72.4)	68 (84.0)	
**Signet-ring cell**					0.704
No Signet-ring cells	92 (62.6)	112 (68.7)	116 (60.4)	51 (62.2)	
Partial signet-ring cells	45 (30.6)	40 (24.5)	63 (32.8)	27 (32.9)	
Signet-ring cell carcinoma	10 (6.8)	11 (6.7)	13 (6.8)	4 (4.9)	
**Pathological T-stage**					0.571
T3-T4	126 (85.7)	141 (86.5)	164 (85.4)	75 (91.5)	
T1-T2	21 (14.3)	22 (13.5)	28 (14.6)	7 (8.5)	
**Pathological N-stage**					0.823
N0	51 (34.7)	50 (30.7)	62 (32.3)	24 (29.3)	
N0-N3	96 (65.3)	113 (69.3)	130 (67.7)	58 (70.7)	
**Metathesis**					0.162
Negative	140 (95.2)	153 (93.9)	189 (98.4)	78 (95.1)	
Positive	7 (4.8)	10 (6.1)	3 (1.6)	4 (4.9)	
**Pathological stage**					0.346
I	28 (19.0)	29 (17.8)	35 (18.2)	9 (11.0)	
II	35 (23.8)	39 (23.9)	39 (20.3)	17 (20.7)	
III	77 (52.4)	85 (52.1)	115 (59.9)	52 (63.4)	
IV	7 (4.8)	10 (6.1)	3 (1.6)	4 (4.9)	
**Surgical margin**					0.234
Negative	144 (98.0)	156 (95.7)	187 (97.4)	82 (100.0)	
Positive	3 (2.0)	7 (4.3)	5 (2.6)	0 (0.0)	

**Figure 2:**
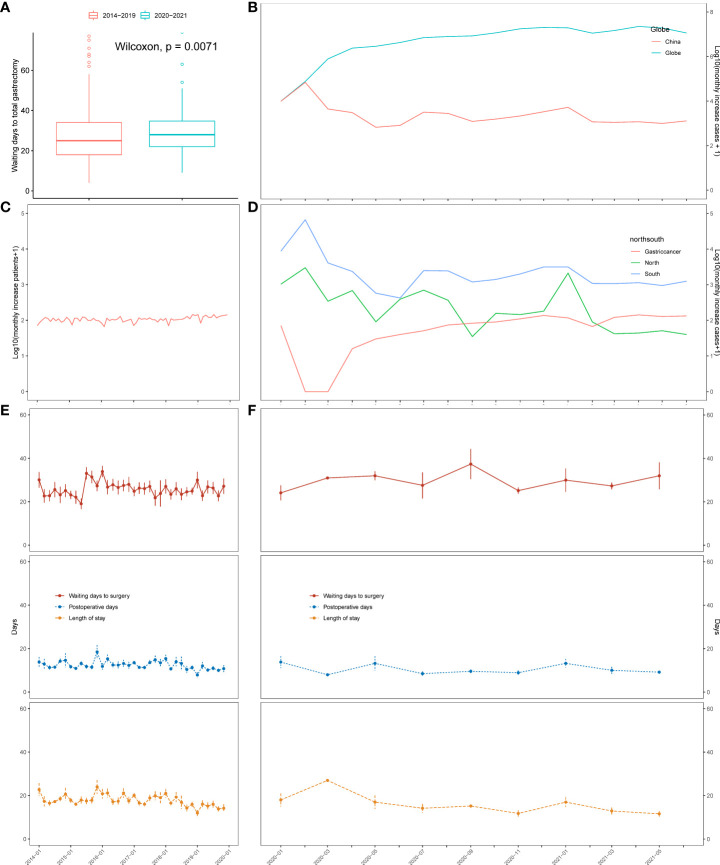
Changes in the severity of the pandemic, number of patients, and the waiting time in days for total gastrectomy based on time series analysis. **(A)** Boxplot of waiting time for total gastrectomy before and after 2020. **(B)** Monthly rise in COVID-19 cases in China and across the globe. **(C)** Changes in the number of surgical cases over time from 2014 to 2019. **(D)** Changes in the number of COVID-19 cases in north China (blue) and south China (green). Number of surgical cases (red) over time from 2020 to 2021. **(E)** Changes in the waiting time for total gastrectomy, length of stay, and postoperative days over time from 2014 to 2019. **(F)** Changes in the waiting time for total gastrectomy, length of stay, and postoperative days over time from 2020 to 2021.

The pandemic has affected and altered the clinical landscape of diagnosis and admission and has prolonged the preoperative waiting time for patients with gastric cancer. **Figure 2A** shows a statistically significant difference in the waiting time for surgery between patients in the pre- and pandemic periods. The median preoperative waiting time before the pandemic was 25.00 days (interquartile range [IQR]: 18.00–34.00), and the median pandemic preoperative waiting time was 28.00 days (IQR: 22.00–34.75). These data show a slight prolongation in the post-pandemic waiting time compared to the pre-pandemic waiting time (*p* = 0.0071). **Figure 2B** shows a line graph of the number of new COVID-19 cases globally and in China from January 2020 to June 2021, when the last patient was admitted. We observed a spike in domestic COVID-19 cases between January and April 2020. After that, compared with the constant reporting of new cases worldwide, the situation in China was relatively under control. The number of new monthly cases gradually decreased and remained stable. **Figures 2C–F** show the admission pattern concerning the number of patients with gastric cancer and the perioperative days of TG patients before and during the COVID-19 pandemic. Overall, from 2014 to 2019, the number of patients who underwent gastrectomy in our hospital was stable, and the monthly median number of gastric cancer patients who underwent gastrectomy was 105.5 (IQR: 96.75–116.25, **Figures 2C**). However, during the pandemic, we observed that the number of patients with gastric cancer treated per month significantly decreased (median 77.50, IQR: 41.75–119.00, *p* = 0.3953, **Figures 2D**). From 2014 to 2021, there was a significant decrease in the number of gastrectomy patients treated per Spring Festival month (January to February, the Chinese New Year holiday) vs. non-Spring Festival months (March to December) (Spring Festival months, median 77.50, IQR: 69.75–90.75; non-Spring Festival months, median patient number: 109.00, IQR: 97.25–119.75, *p* = 0.0004055, **Figures 2C, D**). From 2014 to 2019, compared with the non-Spring Festival months, the number of gastrectomy patients treated from January to February each year was lower (Spring Festival months, median 82.50, IQR: 70.00–90.75; non-Spring Festival months, median 109.00, IQR: 100.00–118.25; *p* = 0.0001725, **Figure 2C**). Meanwhile, we observed no significant difference in the number of patients in the Spring Festival months compared to the non-Spring Festival months during the pandemic (*p* = 0.3953). In addition, the number of COVID-19 cases in northern and southern China was not a good predictor of the trend of patient admissions (**Figure 2D**). Furthermore, we showed dynamic changes in waiting time, length of stay, and POD for TG patients before (**Figure 2E**) and after 2020 (**Figure 2F**). The waiting times during the Spring Festival and non-Spring Festival were not significantly different (*p* = 0.314). Compared with the pre-pandemic period, the post-pandemic POD (before pandemic, median 11.00, IQR: 9.00–13.75; during pandemic, median 9.00, IQR: 8.00–11.00; *p* < 0.001) and LOS (before pandemic, median 16.00, IQR: 14.00–21.00; during pandemic, median 13.00, IQR: 10.00–16.00; *p* < 0.001) showed a significant decrease.

### 3.2 Association between clinicopathological factors and preoperative waiting delay for patients underwent TG

Since there are no SARS-CoV-2 infected cases in our study, no SARS-CoV-2 infection bias was introduced when all patients were included to study the short-term effects of the prolonged waiting time. To summarize, the number of patients with a preoperative waiting time to TG of ≤ 14 days, 15–30 days, and > 30 days was 243 (41.6%), 211 (36.1%), and 130 (22.3%), respectively. To investigate the effect of a prolonged waiting time on pathological status, we divided the entire cohort into prolonged and short waiting groups using 30-days as the cut-off value. There was no difference in the clinical baseline between the two groups of patients (**Table 2**) and that of 82 patients during the pandemic (**Supplementary Table 1**), except for neoadjuvant therapy, which was statistically different between the short waiting group and the prolonged waiting group (*p* < 0.001, all patients; *p* = 0.012, patients during the pandemic). There was no evidence that the prolonged group was in poorer condition. Furthermore, no progression of pathological tumor stage, higher nodal staging, or higher positivity rate of surgical margin status was observed in patients with prolonged preoperative waiting times (**Table 3**, all patients; **Supplementary Table 1**, patients during the pandemic). In addition, we included SA patients with pStage III–IV cancer and the neoadjuvant patients with ypStage III–IV cancer to study the delay’s effect on the short-term outcomes of patients with a higher disease stage. A higher proportion of stage III-IV patients in the delayed surgery group had tumors in the central/upper part of the stomach, and this was associated with a need for additional endoscopy to determine the appropriate surgical margins (*p* = 0.027, **Supplementary Table 2**). Similarly, patients who received neoadjuvant therapy had higher preoperative delay rates (*p* < 0.001, **Supplementary Table 2**). From the above analyses, we found that neoadjuvant therapy was a strong bias factor associated with longer waiting times. We also conducted a subgroup analysis to determine whether the patients received neoadjuvant therapy or not, and similar negative results were observed in the neoadjuvant (**Supplementary Table 3**) and SA subgroups (**Supplementary Table 4**).

**Table 2:** Clinical characteristics of patients according to the waiting time for total gastrectomy.

Variables	Waiting days		P-values
	≤30 days	>30 days	
**COVID-19 cases**	0(0.0)	0(0.0)	
**Age year, mean (SD)**	56.20 (11.22)	57.05 (10.21)	0.385
**Age**			0.709
< 65 years	320 (80.0)	144 (78.3)	
≥ 65 years	80 (20.0)	40 (21.7)	
**Sex**			1
Male	268 (67.0)	123 (66.8)	
Female	132 (33.0)	61 (33.2)	
**Drinking**			0.836
No	240 (60.0)	108 (58.7)	
Yes	160 (40.0)	76 (41.3)	
**Comorbidity**			0.816
No	275 (68.8)	124 (67.4)	
Yes	125 (31.2)	60 (32.6)	
**Neoadjuvant therapy**			< 0.001
Yes	91 (22.8)	115 (62.5)	
No	309 (77.2)	69 (37.5)	

**Table 3:** Pathological characteristics of patients according to the waiting time for total gastrectomy.

Variables	Waiting days		P-values
	≤30 days	> 30 days	
**Tumor Location**			0.123
Middle/Lower	246 (61.5)	100 (54.3)	
Upper	154 (38.5)	84 (45.7)	
**Size cm, mean (SD)**	5.96 (3.40)	6.08 (3.43)	0.713
**Lauren type**			0.924
Intestinal	86 (23.2)	40 (23.8)	
Mixed	94 (25.4)	40 (23.8)	
Diffuse	190 (51.4)	88 (52.4)	
**Bormann type**			0.921
0-1	66 (18.5)	24 (17.6)	
2-4	290 (81.5)	112 (82.4)	
**Differentiation**			1.000
Poorly differentiated	350 (87.5)	161 (87.5)	
Well differentiated	50 (12.5)	23 (12.5)	
**Vessel invasion**			0.305
Negative	163 (41.7)	84 (46.7)	
Positive	228 (58.3)	96 (53.3)	
**Nerve invasion**			0.884
Negative	109 (27.7)	52 (28.7)	
Positive	284 (72.3)	129 (71.3)	
**Signet-ring cell**			0.767
No Signet-ring cells	252 (63.0)	119 (64.7)	
Partial signet-ring cells	120 (30.0)	55 (29.9)	
Signet-ring cell carcinoma	28 (7.0)	10 (5.4)	
**Pathological T-stage**			0.184
T3-T4	341 (85.2)	165 (89.7)	
T1-T2	59 (14.8)	19 (10.3)	
**Pathological N-stage**			0.494
N0	124 (31.0)	63 (34.2)	
N1-N3	276 (69.0)	121 (65.8)	
**Metastasis**			1.000
M0	384 (96.0)	176 (95.7)	
M1	16 (4.0)	8 (4.3)	
**Pathological stage**			0.819
I	69 (17.2)	32 (17.4)	
II	85 (21.2)	45 (24.5)	
III	230 (57.5)	99 (53.8)	
IV	16 (4.0)	8 (4.3)	
**Surgery margin**			1.000
Negative	390 (97.5)	179 (97.3)	
Positive	10 (2.5)	5 (2.7)	

### 3.3 Short-term complications of patients with preoperative waiting delay or without preoperative delay

We assessed complications documented in the medical records, extreme values of laboratory results, and POD to study the effect of the delays on short-term complications. Not all complications were well-documented in the patients’ medical records. CD grade I complications and transient adverse events were often missing, and the comprehensive effect of individual patients cannot be fully described in documented complications using the CD grading system. Thus, objective indices can be a supplement to remarking on short-term outcomes, such as length of stay, POD, results of imaging, and laboratory examinations (14, 15).

#### 3.3.1 Analyses of CD classification of complications in medical records

CD grades II–IV complications are shown in **Table 4**. The most common complications were gastrointestinal motility disorders. **Table 5** shows the most common complication type in patients with CD grades III–IV cases: anastomotic leakage. In patients with prolonged waiting times, we did not observe worse short-term complication rates (grade II–IV: 15% vs. 19%, *p* = 0.27; grade III–IV: 7.3% vs. 9.2%, *p* = 0.51, short waiting group vs. prolonged waiting group).

**Table 4:** A comparison of the complications of cases with grades II–IV between the delayed and the short waiting groups.

Complication names	Waiting days		P-values
	≤ 30 days	> 30 days	
**All Clavien-Dindo grades II–IV, n (%)**	60 (15)	35 (19)	0.27
**Anastomotic leakage, n (%)**	18 (4.5)	6 (3.3)	0.63
**Anastomotic reflux, n (%)**	1 (0.2)	2 (1.1)	0.49
**Gastrointestinal motility disorder, n (%)**	17 (4.2)	8 (4.3)	1.00
**Wound infection, n (%)**	5 (1.2)	3 (1.6)	1.00
**Intraperitoneal infection, n (%)**	11 (2.8)	6 (3.3)	0.94
**Duodenal stump fistula, n (%)**	3 (0.8)	2 (1.1)	1.00
**Postoperative bleeding, n (%)**	3 (0.8)	4 (2.2)	0.29
**Pleural effusion, n (%)**	1 (0.2)	1 (0.5)	1.00
**Anastomotic stenosis, n (%)**	1 (0.2)	2 (1.1)	0.49

**Table 5:** A comparison of the complications of cases with grades III–IV between the delayed and short waiting groups.

Complication names	Waiting days		P-values
	≤ 30 days	> 30 days	
**All Clavien-Dindo grades III–IV, n (%)**	29 (7.3)	17(9.2)	0.51
**Anastomotic leakage, n (%)**	11 (2.8)	3 (1.6)	0.60
**Anastomotic reflux, n (%)**	1 (0.2)	1 (0.5)	1.00
**Gastrointestinal motility disorder, n (%)**	7 (1.8)	3 (1.6)	1.00
**Wound infection, n (%)**	1 (0.2)	1 (0.5)	1.00
**Intraperitoneal infection, n (%)**	2 (0.5)	1 (0.5)	1.00
**Duodenal stump fistula, n (%)**	2 (0.5)	0 (0.0)	0.84
**Postoperative bleeding, n (%)**	3 (0.8)	4 (2.2)	0.29
**Pleural effusion, n (%)**	1 (0.2)	1 (0.5)	1.00
**Anastomotic stenosis, n (%)**	1 (0.2)	2 (1.1)	0.49

#### 3.3.2 Analyses of minimal or maximal postoperative laboratory results

**Figure 3:**
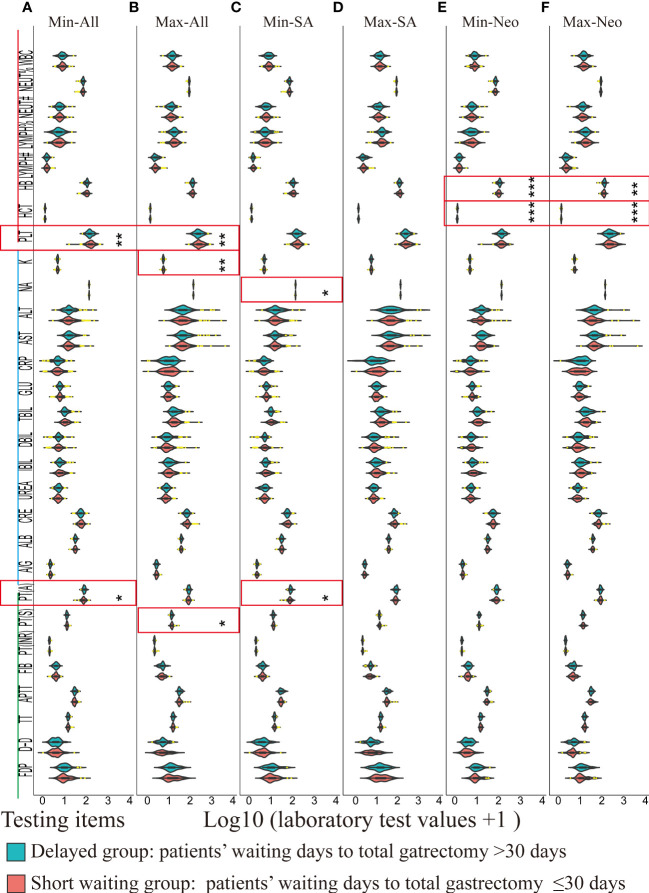
A violin plot of the laboratory test results of routine blood test (red), biochemical tests (blue), and coagulation tests (green) between the delayed (blue green) and the short waiting groups (light red). Laboratory values of outliers (1.5 times greater than or less than the interquartile range) were marked in yellow. Significantly different items between the delayed and the short waiting groups were marked in red rectangles. * *p* < 0.05, ** *p* < 0.01, *** *p* < 0.001. Min: Minimal postoperative laboratory values. Min: Maximal postoperative laboratory values. SA: Surgery alone, Neo: Neoadjuvant therapy plus surgery. WBC: Leukocyte count; NEUT%: Percentage of neutrophils; NEUT#: Absolute Neutrophil count; LYMPH%: Percentage of lymphocytes; LYMPH#: Lymphocyte absolute count; HB: Hemoglobin; HCT: Hematocrit; PLT: platelet count; K: Potassium; NA: Sodium; ALT: Alanine aminotransferase; AST: Aspartate aminotransferase; CRP: C-reactive protein; GLU: Blood sugar; TBIL: Total bilirubin; DBIL: Direct (conjugated) bilirubin; IBIL: Indirect (free) bilirubin; UREA: Urea; CRE: Creatinine; ALB: albumin; A/G: Albumin/globulin ratio; PT(A): Plasma prothrombin activity; PT(S): Plasma prothrombin time; PT(INR): International normalized ratio of prothrombin time; FIB: Fibrinogen; APTT: Activated partial thromboplastin time; TT: Thrombin time; D-D: Plasma D-dimer; FDP: Fibrinogen degradation products; **(A)** Violin plots of minimal postoperative laboratory results of all patients between the delayed and short waiting groups. **(B)** Violin plots of maximal postoperative laboratory results of all patients between the delayed and short waiting groups. **(C)** Violin plots of minimal postoperative laboratory results of SA patients between the delayed and short waiting groups. **(D)** Violin plots of maximal postoperative laboratory results of SA patients between the delayed and short waiting groups. **(E)** Violin plots of minimal postoperative laboratory results of the neoadjuvant patients between the delayed and short waiting groups. **(F)** Violin plots of maximal postoperative laboratory results of the neoadjuvant patients between the delayed and short waiting groups.

Since changes in laboratory results within the normal ranges carry no clinical significance, the extreme (minimal or maximal) postoperative values of the key indicators in routine blood tests, biochemical tests, and coagulation tests in the laboratory information management system were used to indicate the effects in the delayed group compared to those in the short waiting group. The postoperative minimal laboratory results for all patients are shown in **Figure 3A**, and the maximal laboratory results are illustrated in **Figure 3B**. The minimal postoperative prothrombin time activity was lower in the short waiting group among all patients (short waiting group: median 1.89, IQR 1.84–1.93; delayed group: median 1.91, IQR 1.88–1.95; the short waiting group vs. the prolonged group, *p* = 0.012, **Figure 3A**) and in the SA patients (short waiting group: median 1.88, IQR 1.83–1.92; delayed group: median 1.91, IQR 1.89–1.95; the short waiting group vs. the prolonged group, *p* = 0.033, **Figure 3C**). Hemoglobin and hematocrit levels between the two groups showed no significant differences in all patients (**Figures 3A, B**) or SA patients (**Figures 3C, D**). However, patients who received neoadjuvant chemotherapy in the delayed group had a significantly higher postoperative minimal hemoglobin level than those in the short waiting group (median: 2.01, IQR 1.96–2.05 vs. median: 2.04 IQR 1.98–2.08, the short waiting group vs. the prolonged waiting group, *p* = 0.00095), and the hematocrit values followed the same trend (median: 0.11, IQR 0.10–0.12, vs. median: 0.12, IQR 0.11–0.13, *p* = 0.00038, the short waiting group vs. the prolonged waiting group, **Figure 3E**). Higher maximal postoperative hemoglobin (*p* = 0.0015) and hematocrit (*p* = 0.00065) levels were also observed in patients who underwent delayed surgery after neoadjuvant chemotherapy (**Figure 3F**). The minimal postoperative platelet count was lower in the delayed group (median: 2.16; IQR: 2.09–2.24) than in the short waiting group (median: 2.21; IQR: 2.10–2.30, *p* = 0.0024, **Figure 3A**). The values of the maximal platelet showed a similar tendency (delayed group: median 2.37, IQR 2.28–2.49; short waiting group: median 2.41, IQR 2.31–2.54, the short waiting group vs. the prolonged waiting group, *p* = 0.0087, **Figure 3B**); however, the conclusion was not supported by the SA and neoadjuvant subgroups. This suggests that treatment is a potential confounding factor (**Figures 3C–F**). Other laboratory test results did not show significant differences with support in more than one cohort (**Supplementary Datas 1**–**6**).

#### 3.3.3 Analyses of POD

It has been proven that POD is an objective indicator for short-term outcomes, which are closely associated with postoperative complications. Prolongation of POD was associated with a poor short-term prognosis, except for those who experienced sudden death. In the univariable logistic regression analyses of the risk factors for longer POD, smokers compared to non-smokers (OR 1.62, 95% CI: 1.07–2.46, *p* = 0.02) were associated with a longer postoperative stay. However, both the univariable and multivariable-adjusted logistic regression model analyses showed that prolongation of waiting time to TG was not associated with an increased risk of longer POD (univariable: OR 1.09, 95% CI 0.80–1.49, *p* = 0.59; multivariable: OR 1.10, 95% CI 0.78–1.55, *p* = 0.59; **Table 6**).

**Table 6:** Univariable and multivariable logistic regression models of the risk factors for prolonged postoperative stay.

Variable	Reference	Univariable		Multivariable Adjusted*	
		OR [95% CI]	P-values	OR [95% CI]	P-values
Waiting time	Delayed vs. Short	1.09 [0.80–1.48]	0.59	1.10 [0.78–1.55]	0.59
Sex	Male vs. Female	1.40 [0.88–2.21]	0.15	1.12 [0.66–1.90]	0.67
Age	Odds per year	1.01 [0.99–1.03]	0.53	1.00 [0.98–1.02]	0.96
Tumor site	Proximal vs. middle or distal	1.33 [0.87–2.01]	0.18	1.26 [0.81–1.96]	0.31
pT	Advanced vs. early	1.32 [0.82–2.13]	0.25	1.32 [0.77–2.26]	0.31
pN	Positive vs. Negative	1.12 [0.72–1.76]	0.61	1.02 [0.71–1.45]	0.92
Smoking	Yes vs. No	1.62 [1.07–2.46]	0.02	1.56 [0.98–2.47]	0.06
Treatment	Neo vs. SA	1.01 [0.65–1.56]	0.96	0.88 [0.54–1.42]	0.59

*The multivariable-adjusted model included pre-specified potential confounding factors; age, sex, tumor site, pathological tumor stage, and pathological node stage, smoking status, treatment.

OR, odds ratio; CI, confidence interval; Neo, Neoadjuvant therapy plus surgery; SA, surgery alone; pT, pathological tumor stage; pN, pathological node stage.

## 4 Discussion

In this dynamic display of the admission patterns of patients with gastric cancer and TG between the COVID-19 and pre-COVID-19 periods, we found a slight increase in the waiting time for gastric cancer surgery during the COVID-19 period. However, using electronic medical records and laboratory test results, we found no significant difference in short-term postoperative outcomes between the delayed and short waiting groups.

The COVID-19 pandemic has dramatically affected people worldwide, especially those with cancer. Patients had diagnoses missed and delayed treatments due to various factors, including health systems being under pressure and the risk of SARS-CoV-2 infection (16–19). Moreover, we observed a prominent difference in admission patterns before and during the COVID-19 period. Many factors resulted in prolonged preoperative waiting times before the COVID-19 pandemic, such as holidays, underlying diseases, diagnosis level of the hospital at the time, and rotation rate of hospital beds (20, 21). However, the delay caused by the COVID-19 pandemic made the previous experience of delayed surgery related to waiting time no longer as effective as before, which varied in different countries with different policies and severity of the local pandemic (22–24). In this study, a significant decrease in the number of patients was observed in the first two months in 2020 and 2021, which was related to the disruption caused by the nationwide pandemic in 2020 and the local outbreak in 2021, which far exceeded the effect of the holiday delay itself. A country’s prevention policy and the number of new COVID-19 cases had complex, dynamic, and nonlinear effects on admission patterns during the COVID-19 period. European countries have adopted herd immunity and have ended restrictions after vaccination. Although cancer treatment is not directly restricted, serious medical resource panic during the COVID-19 surge led to long-term delays. However, a dynamic COVID-19 control strategy was employed in South Korea and China, which ensured low levels of COVID-19 cases most of the time. An important measure of the zero-COVID strategy was using universal screening for patient admissions and intensive screening from median-to high-risk regions with local outbreaks to mitigate the spread of COVID-19. Except for the suspension of hospital admissions during the initial stage of the pandemic, the prolonged waiting time was slight thereafter (25). Policy-related preoperative screening ensured a significantly lower impact on the waiting times compared to the medical resources panic caused by the outbreak (6). With continued mutation and prevalence of the virus, this slightly prolonged waiting time could persist for the next few years (8, 16).

Longer preoperative waiting times led to paradoxical results based on the different cancer types and waiting times. Although some studies have reported that a long-term delay may result in higher recurrence rates of cancers, the effect of a slight delay on gastric cancer remains undetermined because of different cohorts and cut-off standards for the delay. Studies in the Netherlands showed that waiting time for any treatment (neoadjuvant chemotherapy or gastrectomy) was not associated with lower overall survival (26). A study in Asia reported that it was safe to wait for surgery for one–two more weeks (21, 27). Since there are no SARS-CoV-2 infected cases in our study, no SARS-CoV-2 infection bias was introduced. We did not observe a significant difference in complications between the delayed and short waiting groups, which supported the fact that a dynamic increase in waiting time of one–two weeks did not increase the short-term complication rates of the patients. Additionally, several studies have claimed that the short-term complication rates in patients mainly depend on the surgeon’s skill (20, 28). Contrary to the popular belief that delayed surgery may cause more cancer-related consumption of clotting factors, there was a lower minimal postoperative prothrombin time activity in patients in the short waiting group. Furthermore, we found that the short waiting period group, who also underwent neoadjuvant therapy, had more severe blood loss. These patients had less time to fully recover from temporary myelosuppression and coagulopathy after the cessation of the chemotherapy. However, such changes in the volume of blood loss do not result in a significantly higher rate of complications. Prolonged hospital stay was directly related to more complications and hospital costs, except for patients who died suddenly (29–32). Compared with the pre-pandemic values, the post-pandemic period showed a decrease in length of stay and POD, which might be related to improved surgical quality and longer preparation before TG (33–35). A prolonged preoperative waiting time did not lead to more complications. In contrast, a longer preoperative preparation time leads to a shorter postoperative stay (20).

This study had several limitations. First, our cancer hospital has no cases of emergency gastrectomy for primary gastric cancer. Thus, we could not analyze the effect of waiting on the number of patients requiring emergency surgery. This is an intrinsic shortcoming of specialized hospitals. Second, the patient group was selected from a cohort that returned for follow-up three months after surgery. Therefore, no patients with CD grade V (death) were included in this study, and the 90-day mortality rate was zero in this study.

## Data availability statement

The raw data supporting the conclusions of this article will be made available by the authors, without undue reservation

## Ethics statement

The studies involving human participants were reviewed and approved by the Ethics Committee of Cancer Institute and Hospital, Chinese Academy of Medical Sciences. Written informed consent for participation was not required for this study in accordance with the national legislation and the institutional requirements.

## Author contributions

XZ and SD contributed to study conception and manuscript writing and data analysis. SD, XZ, MW, CS, YW, and SW contributed to data collection. YX, CW, WC, LY, BW, YD, MW, CS, and SW contributed to clinical treatment and diagnosis. All authors contributed to the article and approved the submitted version.

## Funding

This work was supported by CAMS Innovation Fund for Medical Sciences (CIFMS No. 2016-I2M-1-001 and No. 2016-I2M-1-007) and National Natural Science Foundation of China (81972314).

## Conflict of interest

The authors declare that the research was conducted in the absence of any commercial or financial relationships that could be construed as a potential conflict of interest.

## Publisher’s note

All claims expressed in this article are solely those of the authors and do not necessarily represent those of their affiliated organizations, or those of the publisher, the editors and the reviewers. Any product that may be evaluated in this article, or claim that may be made by its manufacturer, is not guaranteed or endorsed by the publisher.

## Supplementary material

Supplementary Table 1 Clinicopathological baseline of 82 patients who underwent TG during the COVID-19 pandemic

Supplementary Table 2 Clinicopathological baseline of 353 stage III–IV gastric cancer patients

Supplementary Table 3 Clinicopathological baseline of 206 patients who underwent neoadjuvant chemotherapy plus total gastrectomy

Supplementary Table 4 Clinicopathological baseline of 378 SA patients

Supplementary Data Sheet 1 Minimal postoperative laboratory results of all patients between the delayed and short waiting groups

Supplementary Data Sheet 2 Maximal postoperative laboratory results of all patients between the delayed and short waiting groups

Supplementary Data Sheet 3 Minimal postoperative laboratory results of the SA patients between the delayed and short waiting groups

Supplementary Data Sheet 4 Maximal postoperative laboratory results of the SA patients between the delayed and short waiting groups

Supplementary Data Sheet 5 Minimal postoperative laboratory results of the neoadjuvant patients between the delayed and short waiting groups

Supplementary Data Sheet 6 Maximal postoperative laboratory results of the neoadjuvant patients between the delayed and short waiting groups
