# Supplementary material for: Dynamic monitoring revealed a slightly prolonged waiting time for total gastrectomy during the COVID-19 pandemic without increasing the short-term complications
Source: Front Oncol. 2022 Aug 31;12:944602. doi: 10.3389/fonc.2022.944602 (PMC9471957; doi:10.3389/fonc.2022.944602)
Supplement: Supplementary Table 1 — Clinicopathological baseline of 82 patients who underwent TG during the COVID-19 pandemic [file Table_1.docx]

**Supplementary Table 1**

|  | **Waiting days** | | **P-vlaue** |
| --- | --- | --- | --- |
| **Variables** | **≤ 30 days** | **> 30 days** |  |
| **COVID-19 cases** | 0(0.0) | 0(0.0) | Not applicable |
| **Age year, mean (SD)** | 57.57 (11.58) | 57.52 (11.51) | 0.985 |
| **Age** |  |  | 0.607 |
| < 65 years | 39 (73.6) | 19 (65.5) |  |
| ≥ 65 years | 14 (26.4) | 10 (34.5) |  |
| **Sex** |  |  | 0.181 |
| Male | 33 (62.3) | 23 (79.3) |  |
| Female | 20 (37.7) | 6 (20.7) |  |
| **Drinking** |  |  | 0.183 |
| No | 35 (66.0) | 14 (48.3) |  |
| Yes | 18 (34.0) | 15 (51.7) |  |
| **Comorbidity** |  |  | 0.464 |
| No | 35 (66.0) | 16 (55.2) |  |
| Yes | 18 (34.0) | 13 (44.8) |  |
| **Neoadjuvant therapy** |  |  | 0.012 |
| Yes | 18 (34.0) | 19 (65.5) |  |
| No | 35 (66.0) | 10 (34.5) |  |
| **Tumor Location** |  |  | 0.380 |
| Middle/Lower | 25 (47.2) | 10 (34.5) |  |
| Upper | 28 (52.8) | 19 (65.5) |  |
| **Size cm, mean (SD)** | 5.69 (2.96) | 5.75 (2.94) | 0.933 |
| **Lauren type** |  |  | 0.728 |
| Intestinal | 10 (19.6) | 7 (25.0) |  |
| Mixed | 9 (17.6) | 6 (21.4) |  |
| Diffuse | 32 (62.7) | 15 (53.6) |  |
| **Bormann type** |  |  | 1.000 |
| 0-1 | 9 (17.3) | 4 (15.4) |  |
| 2-4 | 43 (82.7) | 22 (84.6) |  |
| **Differentiation** |  |  | 0.815 |
| Poorly differentiated | 48 (90.6) | 25 (86.2) |  |
| Well differentiated | 5 (9.4) | 4 (13.8) |  |
| **Vessel invasion** |  |  | 0.501 |
| Negative | 18 (34.0) | 7 (24.1) |  |
| Positive | 35 (66.0) | 22 (75.9) |  |
| **Nerve invasion** |  |  | 1.000 |
| Negative | 8 (15.4) | 5 (17.2) |  |
| Positive | 44 (84.6) | 24 (82.8) |  |
| **Signet-ring cell** |  |  | 0.895 |
| No Signet-ring cells | 33 (62.3) | 18 (62.1) |  |
| Partial signet-ring cells | 17 (32.1) | 10 (34.5) |  |
| Signet-ring cell carcinoma | 3 (5.7) | 1 (3.4) |  |
| **Pathological T-stage** |  |  | 1.000 |
| T3-T4 | 48 (90.6) | 27 (93.1) |  |
| T1-T2 | 5 (9.4) | 2 (6.9) |  |
| **Pathological N-stage** |  |  | 0.616 |
| N0 | 17 (32.1) | 7 (24.1) |  |
| N1-N3 | 36 (67.9) | 22 (75.9) |  |
| **Metastasis** |  |  | 0.245 |
| M0 | 52 (98.1) | 26 (89.7) |  |
| M1 | 1 (1.9) | 3 (10.3) |  |
| **Pathological stage** |  |  | 0.279 |
| I | 6 (11.3) | 3 (10.3) |  |
| II | 13 (24.5) | 4 (13.8) |  |
| III | 33 (62.3) | 19 (65.5) |  |
| IV | 1 (1.9) | 3 (10.3) |  |
| **Surgical margin** |  |  | Not applicable |
| Negative | 53 (100) | 29 (100) |  |
| Positive | 0 (0.0) | 0 (0.0) |  |
