# Supplementary material for: Dynamic monitoring revealed a slightly prolonged waiting time for total gastrectomy during the COVID-19 pandemic without increasing the short-term complications
Source: Front Oncol. 2022 Aug 31;12:944602. doi: 10.3389/fonc.2022.944602 (PMC9471957; doi:10.3389/fonc.2022.944602)
Supplement: Supplementary Table 2 — Clinicopathological baseline of 353 stage III–IV gastric cancer patients [file Table_2.docx]

**Supplementary Table 2**

|  | **Waiting days** | | **P-value** |
| --- | --- | --- | --- |
| **Variables** | **≤ 30 days** | **> 30 days** |  |
| **COVID-19 cases** | 0(0.0) | 0(0.0) | Not applicable |
| **Age year, mean (SD)** | 55.90 (11.50) | 56.35 (11.16) | 0.737 |
| **Age** |  |  | 0.630 |
| < 65 years | 198 (80.5) | 83 (77.6) |  |
| ≥ 65 years | 48 (19.5) | 24 (22.4) |  |
| **Sex** |  |  | 1.000 |
| Male | 158 (64.2) | 69 (64.5) |  |
| Female | 88 (35.8) | 38 (35.5) |  |
| **Drinking** |  |  | 0.733 |
| No | 143 (58.1) | 65 (60.7) |  |
| Yes | 103 (41.9) | 42 (39.3) |  |
| **Comorbidity** |  |  | 1.000 |
| No | 167 (67.9) | 72 (67.3) |  |
| Yes | 79 (32.1) | 35 (32.7) |  |
| **Neoadjuvant therapy** |  |  | < 0.001 |
| Yes | 52 (21.1) | 66 (61.7) |  |
| No | 194 (78.9) | 41 (38.3) |  |
| **Tumor Location** |  |  | 0.027 |
| Middle/Lower | 161 (65.4) | 56 (52.3) |  |
| Upper | 85 (34.6) | 51 (47.7) |  |
| **Size cm, mean (SD)** | 6.82 (3.62) | 7.02 (3.22) | 0.632 |
| **Lauren type** |  |  | 0.891 |
| Intestinal | 36 (15.3) | 17 (16.7) |  |
| Mixed | 52 (22.1) | 24 (23.5) |  |
| Diffuse | 147 (62.6) | 61 (59.8) |  |
| **Bormann type** |  |  | 0.877 |
| 0-1 | 8 (3.6) | 4 (4.8) |  |
| 2-4 | 214 (96.4) | 79 (95.2) |  |
| **Differentiation** |  |  | 1.000 |
| Poorly differentiated | 234 (95.1) | 102 (95.3) |  |
| Well differentiated | 12 (4.9) | 5 (4.7) |  |
| **Vessel invasion** |  |  | 0.232 |
| Negative | 56 (23.0) | 31 (29.8) |  |
| Positive | 187 (77.0) | 73 (70.2) |  |
| **Nerve invasion** |  |  | 0.230 |
| Negative | 28 (11.6) | 7 (6.7) |  |
| Positive | 214 (88.4) | 98 (93.3) |  |
| **Signet-ring cell** |  |  | 0.925 |
| No Signet-ring cells | 140 (56.9) | 62 (57.9) |  |
| Partial signet-ring cells | 87 (35.4) | 38 (35.5) |  |
| Signet-ring cell carcinoma | 19 (7.7) | 7 (6.5) |  |
| **Pathological T-stage** |  |  | 0.459 |
| T2 | 2 (0.6) | 1 (0.4) |  |
| T3 | 58 (16.4) | 37 (15.0) |  |
| T4 | 293 (83.0) | 208 (84.6) |  |
| **Pathological N-stage** |  |  | 1.000 |
| N0 | 3 (1.2) | 1 (0.9) |  |
| N1-N3 | 243 (98.8) | 106 (99.1) |  |
| **Metastasis** |  |  | 0.917 |
| M0 | 230 (93.5) | 99 (92.5) |  |
| M1 | 16 (6.5) | 8 (7.5) |  |
| **Pathological stage** |  |  | 0.917 |
| III | 230 (93.5) | 99 (92.5) |  |
| IV | 16 (6.5) | 8 (7.5) |  |
| **Surgical margin** |  |  | 0.786 |
| Negative | 236 (95.9) | 104 (97.2) |  |
| Positive | 10 (4.1) | 3 (2.8) |  |
