# Supplementary material for: Dynamic monitoring revealed a slightly prolonged waiting time for total gastrectomy during the COVID-19 pandemic without increasing the short-term complications
Source: Front Oncol. 2022 Aug 31;12:944602. doi: 10.3389/fonc.2022.944602 (PMC9471957; doi:10.3389/fonc.2022.944602)
Supplement: Supplementary Table 3 — Clinicopathological baseline of 206 patients who underwent neoadjuvant chemotherapy plus total gastrectomy [file Table_3.docx]

**Supplementary Table 3**

|  | **Waiting days** | | **P-value** |
| --- | --- | --- | --- |
| **Variables** | **≤ 30 days** | **> 30 days** |  |
| **COVID-19 cases** | 0(0.0) | 0(0.0) |  |
| **Age year, mean (SD)** | 55.58 (10.65) | 55.90 (9.96) | 0.828 |
| **Age** |  |  | 0.856 |
| < 65 years | 75 (82.4) | 97 (84.3) |  |
| ≥ 65 years | 16 (17.6) | 18 (15.7) |  |
| **Sex** |  |  | 0.734 |
| Male | 62 (68.1) | 82 (71.3) |  |
| Female | 29 (31.9) | 33 (28.7) |  |
| **Comorbidity** |  |  | 0.250 |
| No | 44 (48.4) | 66 (57.4) |  |
| Yes | 47 (51.6) | 49 (42.6) |  |
| **Diseases** |  |  | 0.920 |
| No | 64 (70.3) | 79 (68.7) |  |
| Yes | 27 (29.7) | 36 (31.3) |  |
| **Tumor Location** |  |  | 0.433 |
| Middle/Lower | 51 (56.0) | 57 (49.6) |  |
| Upper | 40 (44.0) | 58 (50.4) |  |
| **Size cm, mean (SD)** | 6.28 (4.08) | 6.09 (3.37) | 0.713 |
| **Lauren type** |  |  | 0.705 |
| Intestinal | 14 (19.2) | 25 (24.5) |  |
| Mixed | 19 (26.0) | 25 (24.5) |  |
| Diffuse | 40 (54.8) | 52 (51.0) |  |
| **Bormann type** |  |  | 0.468 |
| 0-1 | 9 (16.4) | 7 (10.3) |  |
| 2-4 | 46 (83.6) | 61 (89.7) |  |
| **Differentiation** |  |  | 1.000 |
| Poorly differentiated | 79 (86.8) | 99 (86.1) |  |
| Well differentiated | 12 (13.2) | 16 (13.9) |  |
| **Vessel invasion** |  |  | 0.804 |
| Negative | 48 (52.7) | 56 (50.0) |  |
| Positive | 43 (47.3) | 56 (50.0) |  |
| **Nerve invasion** |  |  | 0.100 |
| Negative | 34 (37.4) | 29 (25.7) |  |
| Positive | 57 (62.6) | 84 (74.3) |  |
| **Signet-ring cell** |  |  | 0.604 |
| No Signet-ring cells | 63 (69.2) | 80 (69.6) |  |
| Partial signet-ring cells | 25 (27.5) | 28 (24.3) |  |
| Signet-ring cell carcinoma | 3 (3.3) | 7 (6.1) |  |
| **Pathological T-stage** |  |  | 0.072 |
| T3-T4 | 80 (87.9) | 110 (95.7) |  |
| T1-T2 | 11 (12.1) | 5 (4.3) |  |
| **Pathological N-stage** |  |  | 0.977 |
| N0 | 29 (31.9) | 38 (33.0) |  |
| N1-N3 | 62 (68.1) | 77 (67.0) |  |
| **Metastasis** |  |  | 1.000 |
| M0 | 86 (94.5) | 108 (93.9) |  |
| M1 | 5 (5.5) | 7 (6.1) |  |
| **Pathological stage** |  |  | 0.987 |
| I | 14 (15.4) | 16 (13.9) |  |
| II | 25 (27.5) | 33 (28.7) |  |
| III | 47 (51.6) | 59 (51.3) |  |
| IV | 5 (5.5) | 7 (6.1) |  |
| **Surgical margin** |  |  | 0.647 |
| Negative | 89 (97.8) | 110 (95.7) |  |
| Positive | 2 (2.2) | 5 (4.3) |  |
