# Supplementary material for: Dynamic monitoring revealed a slightly prolonged waiting time for total gastrectomy during the COVID-19 pandemic without increasing the short-term complications
Source: Front Oncol. 2022 Aug 31;12:944602. doi: 10.3389/fonc.2022.944602 (PMC9471957; doi:10.3389/fonc.2022.944602)
Supplement: Supplementary Table 4 — Clinicopathological baseline of 378 SA patients [file Table_4.docx]

**Supplementary Table 4**

|  | **Waiting days** | | **P-value** |
| --- | --- | --- | --- |
| **Variables** | **≤ 30 days** | **> 30 days** |  |
| **COVID-19 cases** | 0(0.0) | 0(0.0) |  |
| **Age year, mean (SD)** | 56.39 (11.39) | 58.97 (10.39) | 0.084 |
| **Age** |  |  | 0.065 |
| < 65 years | 245 (79.3) | 47 (68.1) |  |
| ≥ 65 years | 64 (20.7) | 22 (31.9) |  |
| **Sex** |  |  | 0.316 |
| Male | 206 (66.7) | 41 (59.4) |  |
| Female | 103 (33.3) | 28 (40.6) |  |
| **Smoking** |  |  | 0.908 |
| No | 192 (62.1) | 44 (63.8) |  |
| Yes | 117 (37.9) | 25 (36.2) |  |
| **Drinking** |  |  | 0.795 |
| No | 196 (63.4) | 42 (60.9) |  |
| Yes | 113 (36.6) | 27 (39.1) |  |
| **Comorbidity** |  |  | 0.726 |
| No | 211 (68.3) | 45 (65.2) |  |
| Yes | 98 (31.7) | 24 (34.8) |  |
| **Tumor Location** |  |  | 1.000 |
| Middle/Lower | 195 (63.1) | 43 (62.3) |  |
| Upper | 114 (36.9) | 26 (37.7) |  |
| **Size cm, mean (SD)** | 5.87 (3.17) | 6.05 (3.55) | 0.681 |
| **Lauren type** |  |  | 0.834 |
| Intestinal | 72 (24.2) | 15 (22.7) |  |
| Mixed | 75 (25.3) | 15 (22.7) |  |
| Diffuse | 150 (50.5) | 36 (54.5) |  |
| **Bormann type** |  |  | 0.337 |
| 0-1 | 57 (18.9) | 17 (25.0) |  |
| 2-4 | 244 (81.1) | 51 (75.0) |  |
| **Differentiation** |  |  | 0.769 |
| Poorly differentiated | 271 (87.7) | 62 (89.9) |  |
| Well differentiated | 38 (12.3) | 7 (10.1) |  |
| **Vessel invasion** |  |  | 0.767 |
| Negative | 115 (38.3) | 28 (41.2) |  |
| Positive | 185 (61.7) | 40 (58.8) |  |
| **Nerve invasion** |  |  | 0.172 |
| Negative | 75 (24.8) | 23 (33.8) |  |
| Positive | 227 (75.2) | 45 (66.2) |  |
| **Signet-ring cell** |  |  | 0.287 |
| No Signet-ring cells | 189 (61.2) | 39 (56.5) |  |
| Partial signet-ring cells | 95 (30.7) | 27 (39.1) |  |
| Signet-ring cell carcinoma | 25 (8.1) | 3 (4.3) |  |
| **Pathological T-stage** |  |  | 0.433 |
| T3-T4 | 261 (84.5) | 55 (79.7) |  |
| T1-T2 | 48 (15.5) | 14 (20.3) |  |
| **Pathological N-stage** |  |  | 0.458 |
| N0 | 95 (30.7) | 25 (36.2) |  |
| N1-N3 | 214 (69.3) | 44 (63.8) |  |
| **Metastasis** |  |  | 0.600 |
| M0 | 298 (96.4) | 68 (98.6) |  |
| M1 | 11 (3.6) | 1 (1.4) |  |
| **Pathological stage** |  |  | 0.615 |
| I | 55 (17.8) | 16 (23.2) |  |
| II | 60 (19.4) | 12 (17.4) |  |
| III | 183 (59.2) | 40 (58.0) |  |
| IV | 11 (3.6) | 1 (1.4) |  |
| **Surgical margin** |  |  | 0.374 |
| Negative | 301 (97.4) | 69 (100.0) |  |
| Positive | 8 (2.6) | 0 (0.0) |  |
